# Molecular Characterization of Rice *OsLCB2a1* Gene and Functional Analysis of its Role in Insect Resistance

**DOI:** 10.3389/fpls.2016.01789

**Published:** 2016-12-01

**Authors:** Mahfuj A. Begum, Xiao-Xiao Shi, Ye Tan, Wen-Wu Zhou, Yusuf Hannun, Lina Obeid, Cungui Mao, Zeng-Rong Zhu

**Affiliations:** ^1^State Key Laboratory of Rice Biology, Key Laboratory of Agricultural Entomology, Ministry of Agriculture and Institute of Insect Sciences, Zhejiang UniversityHangzhou, China; ^2^Stony Brook Cancer Center, Department of Medicine, The State University of New York at Stony BrookNew York, NY, USA

**Keywords:** serine palmitoyltransferase, long-chain base, sphingolipids, Arabidopsis, insect resistance, *Myzus persicae*, electrical penetration graph

## Abstract

In plants, sphingolipids, such as long-chain bases (LCBs), act as bioactive molecules in stress responses. Until now, it is still not clear if these lipids are involved in biotic stress responses to herbivore. Herein we report that a rice LCB gene, *OsLCB2a1* encoding a subunit of serine palmitoyltransferase (SPT), a key enzyme responsible for the *de novo* biosynthesis of sphingolipids, plays a critical role in plant defense response to the brown planthopper (BPH) attack and that its up-regulation protects plants from herbivore infestation. Transcripts of *OsLCB2a1* gene in rice seedlings were increased at 4 h, but decreased at 8–24 h after BPH attack. Sphingolipid measurement profiling revealed that overexpression of *OsLCB2a1* in *Arabidopsis thaliana* increased trihydroxylated LCB phytosphingosine (t18:0) and phytoceramide by 1.7 and 1.3-fold, respectively, compared with that of wild type (WT) plants. Transgenic Arabidopsis plants also showed higher callose and wax deposition in leaves than that of WT. Overexpression of *OsLCB2a1* gene in *A. thaliana* reduced the population size of green peach aphid (*Myzus persicae*). Moreover, the electrical penetration graph (EPG) results indicated that the aphids encounter resistance factors while reaching for the phloem on the transgenic plants. The defense response genes related to salicylic acid signaling pathway, remained uplgulated in the *OsLCB2a1*-overexpressing transgenic plants. Our data highlight the key functions of *OsLCB2a1* in biotic stress response in plants.

## Introduction

Sphingolipids, a group of lipids, are essential structural components of the endomembrane system present in a variety of organisms including eukaryotes and bacteria (Lynch and Dunn, 2004; Hannun and Obeid, 2008; Merrill et al., 2009). They are a group of lipids containing a backbone of sphingoid bases (1, 3- dihydroxy- 2-amino- alkane and its derivatives), a set of aliphatic amino alcohols that includes sphingosine. In mammals, sphingolipid metabolites, such as sphingosine-1-phosphate (S1P) and ceramide, also act as signaling and regulatory molecules to regulate stress responses, apoptosis and cell proliferation (Chalfant and Spiegel, 2005; Zauner et al., 2010; Markham et al., 2011; Chen et al., 2012). Like animals, plants contain a variety of sphingolipid metabolites, such as ceramides, glycosylceramides, LCB, LCB phosphates, phosphosphingolipids, and glycosyl inositol phosphorylceramide (Pata et al., 2010; Berkey et al., 2012). Till date thousands of different molecular species of sphingolipid have been identified in a range of plants species. For instance, in Arabidopsis at least 168 different sphingolipids (Markham and Jaworski, 2007) have been found, while 30 different ceramide cores have been reported in rye (*Secale cereale*) leaves (Cahoon and Lynch, 1991). Various enzymes involved in plant sphingolipid metabolism have now been cloned and characterized. Serine palmitoyltransferase (SPT) catalyzes the first step of LCB synthesis, which condenses serine and palmitoyl-CoA to form 3-ketosphinganine in a pyridoxal 5′-phosphate (PLP)-dependent reaction. 3-Ketosphinganine is immediately converted to dihydro- sphingosine (sphinganine) by NADPH-dependent 3-ketosphinganine reductase. Dihydro-sphingosine, in turn, is converted to dihydroceramide, which gives rise to ceramide. The plant SPT, like all known eukaryotic SPTs, is a heterodimer and is composed of LCB1 and LCB2 subunits (Chen et al., 2006). SPT is anchored to the endoplasmic reticulum (Hanada, 2003). Both LCB1 and LCB2 sub-units are conserved in plants and are required for the function of SPT (Chen et al., 2006; Dietrich et al., 2008). Sphinganine is the first LCB produced in plants, which is fully saturated and contains two hydroxyl groups. Sphinganine can be further modified by the addition of a hydroxyl group at C4 to yield phytosphingosine and/or by introduction of double bonds at C4 and C8 to produce other LCBs (Lynch and Dunn, 2004).

In plants, the disruption of sphingolipid metabolism affects plant growth and development (Zheng et al., 2005; Imamura et al., 2007; Chen et al., 2008; Dietrich et al., 2008; Chao et al., 2011; Wu et al., 2015), as well as responses to biotic and abiotic stresses (Ng and Hetherington, 2001; Ng et al., 2001; Lynch and Dunn, 2004; Lynch et al., 2009). S1P and other sphingolipids play important roles in multiple abiotic stress responses, especially in the drought stress response (Ng et al., 2001; Coursol et al., 2003, 2005; Ryan et al., 2007; Worrall et al., 2008; Quist et al., 2009). S1P has been shown to promote stomatal closure in a calcium dependent manner (Ng et al., 2001; Coursol et al., 2005; Xiong et al., 2008). A recent study demonstrated that Arabidopsis alkaline ceramidase (*AtACER*) affects sphingolipid homeostasis and plays important roles in plant development, stress responses, salt tolerance and disease resistance (Wu et al., 2015). Some studies with overexpression transgenic lines and knock-out mutants have showed that enzymes involved in sphingolipid biosynthesis act as important regulators of non-host disease resistance, programmed cell death (PCD) and defense response against pathogen attack (Shi et al., 2007; Takahashi et al., 2009; Alden et al., 2011; Saucedo-Garcia et al., 2011; Wu et al., 2015).

Furthermore, an increase in sphingolipid biosynthesis due to a change in the activity of serine palmitoyltransferase enhances resistance to syringomycin E. that is produced by strains of the plant bacterium *Pseudomonas syringae* pv. *Syringae* (Toume and Tani, 2014). Takahashi et al. (2009) revealed that over-expression of *LCB2* (the function regulating subunit of SPT) in *Nicotiana benthamiana*, resulted in hypersensitive response—like cell death. In *N. benthamiana*, SPTLCB2 subunit is vigorously induced upon infection with the non-host pathogen *Pseudomonas cichorii*, but only a minor and transient induction is observed in response to the host pathogen *P. syringae* pv. *tabaci* (Takahashi et al., 2009). Sphingobase, phytosphingosine (t18:0) was increased in Arabidopsis leaves after inoculation with *P. syringae* in an incompatible interaction that leads to resistance, as compared with the compatible one (Peer et al., 2010). These information suggest that *de novo* synthesis of LCBs plays a role in pathogen defense.

However, little is known about the physiological function of SPT in herbivore resistance in plants. In order to gain a better insight into the role of sphingolipids in plant defense response to insects, we over-expressed a rice LCB gene, *OsLCB2a1* in *A. thaliana*. Overexpression of *OsLCB2a1* resulted in increase of LCBs and ceramides in leaves. Over-expression of this gene in *A*. *thaliana* leads to reduce the population size of *M. persicae*. Moreover, EPG results indicated that the aphids encountered resistance factors while reaching for the phloem on the over-expressing plant. Our results suggest that *OsLCB2a1* is involved in plant defense against herbivore and thus signifies potential implication of SPT in insect resistance.

## Materials and methods

### Plant growth

Seeds of the rice (*Oryza sativa*) genotype Xiushui 11, a *japonica* variety, were sown in a plastic box containing rice nutrient solution (Yoshida et al., 1976) and maintained in a controlled climate room at 26 ± 2°C, 12 h light phase and 80% relative humidity. Arabidopsis (Col-0) seeds were obtained from Arabidopsis Biological Resource Center at Ohio State University. Seeds were surface sterilized with 10% bleach and sown onto half-strength MS medium. To induce germination, seeds were placed at 4°C in the dark for 3 days under high humidity. Twelve to fifteen-day-old seedlings were transferred to pots containing soil mix. Plants were cultivated in a growth chamber under a 10-h photo period/14-h dark at 22°C for vegetative stage and 16-h photoperiod/8-h dark for reproductive stage. Plants were watered twice a week with Hogland's nutrient solution but, no pesticide was applied.

### Insect rearing

Colonies of the brown planthopper (*Nilaparvata lugens*) were maintained on Taichung Native 1 (TN1, an *indica* variety without any resistant gene to herbivores and pathogens) rice seedlings in a controlled climate room at 26 ± 2°C, 12 h light phase and 80% relative humidity. The green peach aphids (GPA, *M. persicae*) were reared in cages on the Chinese cabbage (*Brassica rapa* L.). Aphid rearing was maintained in an acclimatized room with a relative humidity of 60–70%, a temperature of 20 ± 2°C and an 18:6 L: D photoperiod. For all experiments, only apterous aphids were used.

### Generation of transgenic plants

The coding sequence of the *OsLCB2a1* gene was amplified from pMD18T*OsLCB2a1* using *OsLCB2a1*-ORF-F (GCTCTAGAATGGTGAGGCTGCCCTAC) (*XbaI* site underlined) and *OsLCB2a1*-ORF-R (GGGGTACCTTGAAGCTCTTCAGTTTCTCA) (*KpnI* site underlined). After digestion with *XbaI/KpnI*, the coding sequence of the *OsLCB2a1* was ligated into plant binary vector pCAMBIA1300 under the cauliflower mosaic virus 35 S promoter in sense orientation. The resulting recombinant plasmid, designated as pCAMBIA-*OsLCB2a1*, was introduced into *Agrobacterium tumefaciens* strain GV1301. *A. tumefaciens* mediated transformation was used to introduce the transgene *OsLCB2a1* into Columbia-0 (Col-0) by floral deep method (Zhang et al., 2006). Two independent transformants in Col-0, referred to as Line OE1 and Line OE2 were used in further experiments. Transformed progenies were screened on half-strength MS medium supplemented with 0.25 mg/L hygromycin. Homozygous transgenic lines of T3 generation were used for the experiments.

### Quantitative RT-PCR (qPCR)

Total RNA from leaf or seedling samples was extracted using extraction kit (Tiangen, Shanghai, China) and reversely transcribed to cDNA using a PrimeScript RT reagent Kit according to the manufacture's instruction (Takara). Gene-specific primers were designed with Primer-3-Plus software. qPCR was performed in a total volume of 50 μl containing 4 μl cDNA, 1.5 μl of each gene-specific primer (10 mM), 18 μl ddH_2_O and 25 μl SYBR Green Super mix Reagent (BioRad). qPCR was run on an ABI 7500 Real-Time PCR System for one cycle of 95°C for 3 min followed by 40 cycles of 95°C for 10 s, and 60°C for 30 s. Normalized gene expression was calculated by the 2^−ΔΔCT^ method (Livak and Schmittgen, 2001). Primers used in this study are listed in Table S2.

### Thermal asymmetric interlaced (TAIL) PCR

To determine the T-DNA border sequence and insertion position of *OsLCB2a1* gene in Arabidopsis over-expressed plants TAIL PCR (Liu and Whittier, 1995) was done. We used three nested T-DNA border region specific primers LBSP1, LBSP2, LBSP3 together with mixture of three arbitrary degenerate primers AD1, AD2 and AD3. Primers sequences are shown in Table S2.

### Sequence analysis

LCB2 homolog sequences were obtained by BLASTp (http://blast.ncbi.nlm.nih.gov), and Phytozome 10.3 (http://phytozome.jgi.doe.gov) and representative sequences were aligned by CLUSTALW using GENETYX program (Software Development Co., Ltd., Tokyo, Japan). The aligned sequences were used for phylogenetic analysis by Maximum Likelihood (ML) method by MEGA 5.0 software. Accession numbers of different organisms used in this tree contraction in the NCBI and Phytozome Genbank were given in Table S1.

### Measurement of sphingolipids

Measurement of sphingolipids was performed as previously described by Markham and Jaworski (2007) with minor modifications. Briefly, about 600 mg of shoots from 4-week-old WT and over-expressed plants were homogenized. The internal standards (C17baseD-*erythro*-sphingosine and C_12_-Ceramide) were added and extracted with the isopropanol/hexane/water (55:20:25 v/v/v). After incubation at 60°C for 15 min, the supernatants were dried by nitrogen. The dried extracts were de-esterified in 33% methylamine in ethanol/water (7: 3 v/v) for 1 h of incubation at 50°C. The samples were dried under nitrogen, then dissolved in 1 ml of methanol and analyzed by the Shimadzu UFLCXR/Triple-TOF 5600 LC/MS system using an Agilent Eclipse XDB C8 column (50 92.1 mm, 1.8 l m). Peak score responding to the target analytics and internal standards were collected and processed using the Agilent Masshunter Quantitative Analysis software. The components of sphingolipids were determined as described previously (Markham and Jaworski, 2007). Three biological repeats were performed.

### Aphid population growth in tested Arabidopsis plants

No-choice aphid tests were performed with 15 biological replicates per genotype. Synchronized 1-day-old nymphs were used to infest 4-week-old plants with one nymph per plant. Nymphs were transferred to the plants using a fine camel hair brush. The total number of aphids was counted 14 days after infestation.

### Aphid probing and feeding behavior through EPG

The EPG technique (Tjallingii, 1988; Reese et al., 2000) was employed to monitor penetrating and feeding behavior of aphids on over-expressed and WT plants. A gold wire (diameter 20 mm) was attached onto the dorsum of young adult aphids using conductive water-based silver glue. The wired aphid was placed on a 4-week-old plant that was connected to a recording system via a copper electrode in the soil (Tjallingii, 2006). The EPGs were recorded in a 22°C room with constant light for 8 h. At least 15 recordings of individual aphids (one aphid per plant) were obtained for each line. The EPG data were analyzed using the PROBE 3.0 software (Wageningen University, the Netherlands) to distinguish the various waveforms. Waveform C represents the pathway phase, when the aphid stylet is penetrating through the leaf tissue; waveform E2 represents phloem sap ingestion; Waveform F is associated with derailed stylet mechanics or penetration difficulties; and waveform G indicates active uptake of water from the xylem elements.

### Exogenous phytosphingosine experiment

Arabidopsis plants were grown in soil. After 4 weeks the plants were sprayed with 25 μM phytospingosine or methanol as methanol was used to prepare stock solution of phytospingosine. After 8 h rosette leaves were collected and immediately frozen in liquid nitrogen for sphingolipid measurement. Three biological repeats were performed.

### Leaf callose deposition detection

Callose deposition in leaves of the transgenic Arabidopsis plant and WT was visualized as a violet color by staining with aniline blue. Leaves were infiltrated with 5 ml of a solution made of phenol, glycerol, lactic acid, water and 95% ethanol (1:1:1:1:2, v/v). Leaves in solution were incubated in a 65°C bath until they become transparent and then stained with aniline blue. The staining reaction was held in the dark for 4 h. Samples were observed by microscopy under ultraviolet field (Zhang et al., 2014).

### Field emission scanning electron microscopy analysis

To visualize the alteration in cuticular wax content, a Field emission scanning electron microscope was used to analyze the images of Arabidopsis leaves without any form of preparation (Zimmermann et al., 2007). The adaxial side of 4-week-old rosette leaves of OE and WT plants were collected and directly placed onto the stub.

### Statistical analysis

Data were subjected to analysis of variance (ANOVA) and presented as the mean value for each treatment. Statistical analyses were performed using Data Processing System (DPS) statistical software package (Tang and Zhang, 2013; http://www.dpsw.cn; Zhu et al., 2015). A Tukey's test (*P* < 0.05) was performed to evaluate the treatment effects.

## Results

### Cloning and characterization of *OsLCB2a1*

**Figure 1:**
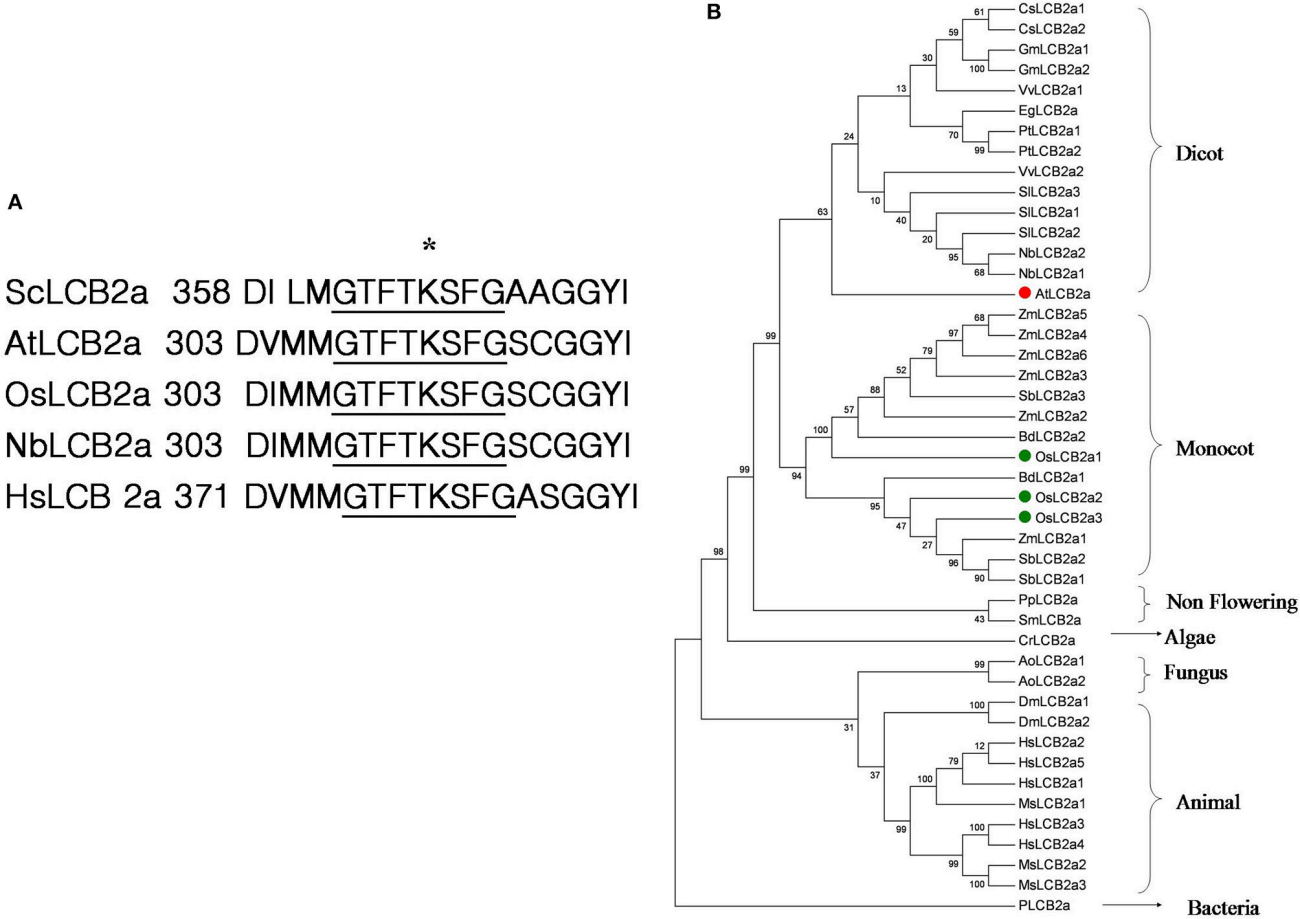
**(A)** Comparison of the pyridoxal 5′-phosphate (PLP)-binding motif of LCB2a. Species are indicated as follows: Nb, *Nicotiana benthamiana*; At, *Arabidopsis thaliana*; Os, *Oryza sativa*; Hs, *Homo sapiens*; Sc, *Saccharomyces cerevisiae*. An asterisk marks the PLP-binding lysine residue. **(B)** Phylogenetic tree of LCB2a homolog in different organism.

To investigate the possible function of sphingolipid in insect resistance response, we cloned a gene (Os01g70380) from *O. sativa* encoding the LCB2a subunit of SPT, designated as *OsLCB2a1*. The full-length cDNA of the *OsLCB2a1* gene was amplified by RT-PCR. Sequence analysis showed that *OsLCB2a1* contains a 1470 bp open reading frame and encodes a protein of 489 amino acids which shares high identity with LCB2a from other plants. To identify LCB2a homology, we used BLASTp to conduct sequence similarity searches using the sequence of *A. thaliana* (*AtLCB2a*, AT5G23670). CLUSTALW alignment of the amino acid sequence of *OsLCB2a1* with LCB2a from various organisms showed the presence of a KBL like domain (Figure S1) and a conserved GTFTKSFG motif (amino acid positions 307–314) corresponding to the known PLP-binding site, which is commonly found in members of the α-oxoamine synthase subfamily, such as 5-aminolevulinic acid synthase, 2-amino-3-ketobutyrate CoA ligase, and 8 amino-7-oxononanoate synthase (Figure 1A; Hanada, 2003). Phylogenetic analysis showed that LCB2a can be divided into seven categories: dicot, monocot, non-flowering plant, algae, fungi, animal, and bacteria and that *OsLCB2a1* belongs to monocot branch (Figure 1B). There were three copies of LCB2a in rice (Figure 1B). *OsLCB2a1* has 90, 84, and 82% identity to *Zea mays* SPT2, *N. banthamiana* SPT, and *A. thalian*a LCB2a, respectively.

### *OsLCB2a1* expression is up-regulated under the brown planthopper (BPH) infestation

**Figure 2:**
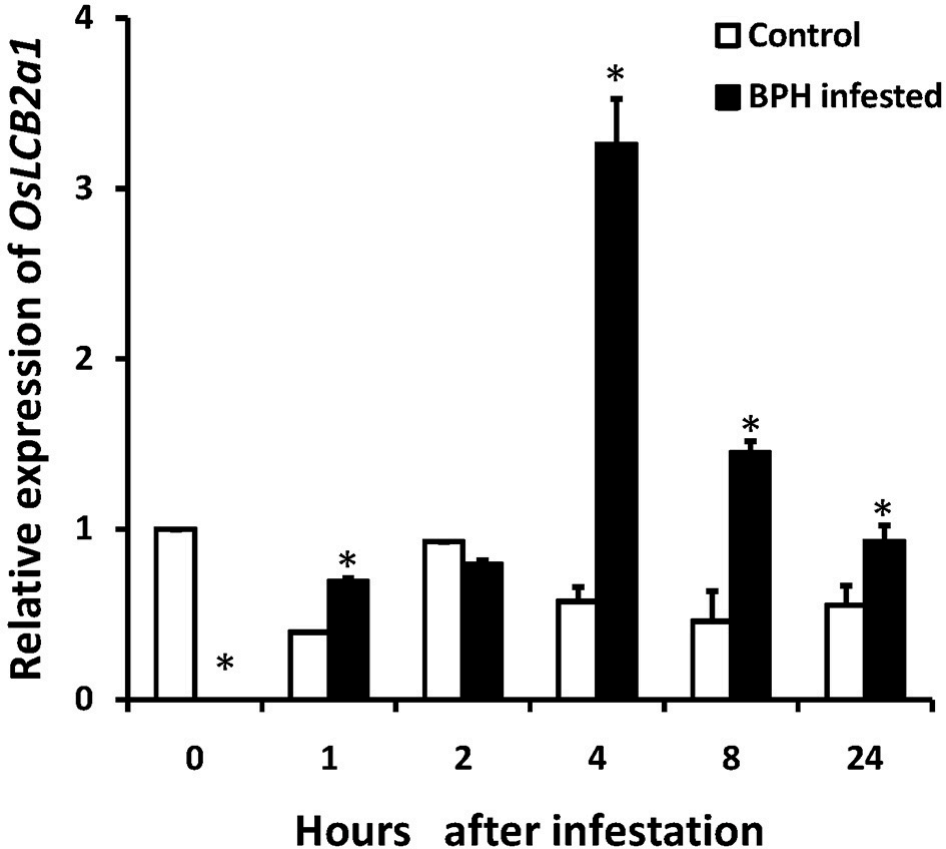
**Expression of ***OsLCB2a1*** under BPH attack**. Two weeks rice seedlings were infested with 3rd instar BPH nymph (20 nymphs/ seedling). Total RNA was isolated from rice seedlings infested by BPH at 0, 1, 2, 4, 8, 24 h and reversely transcribed into cDNA used as template in quantitative RT-PCR analysis. Values are mean ± SE (*n* = 5). The asterisks indicate statistically significant differences between the treatments (One way ANOVA followed by Tukey's tests, *P* < 0.05).

BPH is one of the most important rice pests in Asia with piercing and sucking mouthpart that are needlelike. They suck plant sap during their feeding on rice tissues. We performed the experiment on the effect of BPH infestation on *OsLCB2a1* inducement. The experimental results revealed that *OsLCB2a1* transcripts in rice seedling were increased with time, reaching a peak at 4 h after that declined at 8–24 h under BPH attack (Figure 2).

### Overexpression of *OsLCB2a1* increases sphingolipids in Arabidopsis plants

To examine *OsLCB2a1* function, we transferred the *OsLCB2a1* gene into WT Arabidopsis (Col-0) and generated the over-expression (OE) lines. RT-PCR result showed that the *OsLCB2a1*-OE plants but not the WT plants amplified a DNA band corresponding to the *OsLCB2a1 trans*-gene (Figure S2). Quantification of *OsLCB2a1* expression through qRT-PCR in 4-week-old leaves of Arabidopsis plants revealed that the gene expression was increased by a 33-fold in the OE plant compared to the WT plant (Figure S3). The T-DNA border sequence and insertion position of transgenic plants were confirmed by TAIL PCR (Liu and Whittier, 1995). The insertion site located upstream 1 kb of AT5G56900 and downstream 2.7 kb of AT5G56890, without any annotated genes broken in the OE plant (Figure S4).

**Figure 3:**
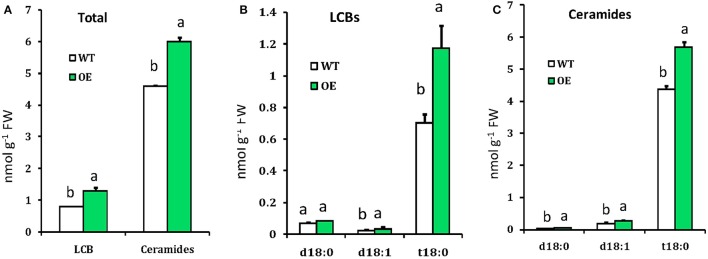
**Measurement of sphingolipids in WT and OE plants. (A)** Total LCBs and ceramides in indicating plants. **(B)** Free LCBs composition. **(C)** LCB moiety distribution of ceramide species. Values represent means ± SE from three independent experiments. Different letters indicate significant differences (*P* < 0.05) between the treatments.

As SPT is the enzyme that catalyzes the first step for sphingolipid biosynthesis (Gable et al., 2000; Breslow and Weissman, 2010), we hypothesized that its overexpression could change the sphingolipid profile in Arabidopsis. Sphingolipids were extracted from leaves of 4-week-old WT and OE plants and quantitatively analyzed by LC-MS/MS. Phytosphingosine (t18:0), sphingosine (d18:1), sphinganine (d18:0), different ceramides and phytoceramides were identified by the retention time of the corresponding standards. As expected, LCBs and ceramides were increased in the OE plants compared with the WT (Figure 3). LCB composition of Arabidopsis leaves was similar to those reported previously for other *Solanacea*, such as tobacco and tomato (Sperling et al., 2005; Markham et al., 2006; Buré et al., 2014; Wu et al., 2015), in which trihydroxylated LCBs such as phytosphingosine was more abundant than the dihydroxylated LCBs sphinganine and sphingosine (Figure 3B). Phytoceramide (t18:0) was more abundant than ceramide (d18:1) and hydroxyceramide (d18:0), these findings were similar to Wu et al. (2015). Comparing ceramides with LCB moieties, we found a higher level of phytoceramides in OE plants than that in the WT plants (Figure 3C). Thus, the quantitative sphingolipid profiling showed that the OE plants accumulated more long-chain bases (1.3 nmolg^−1^FW) and ceramides (6.0 nmolg^−1^FW) compared to WT (LCBs 0.8 nmolg^−1^FW and ceramide 4.6 nmolg^−1^FW). These data suggested that overexpression of *OsLCB2a1* in Arabidopsis increase the level of sphingolipids.

### Overexpression of *OsLCB2a1* increases callose and wax deposition in Arabidopsis

**Figure 4:**
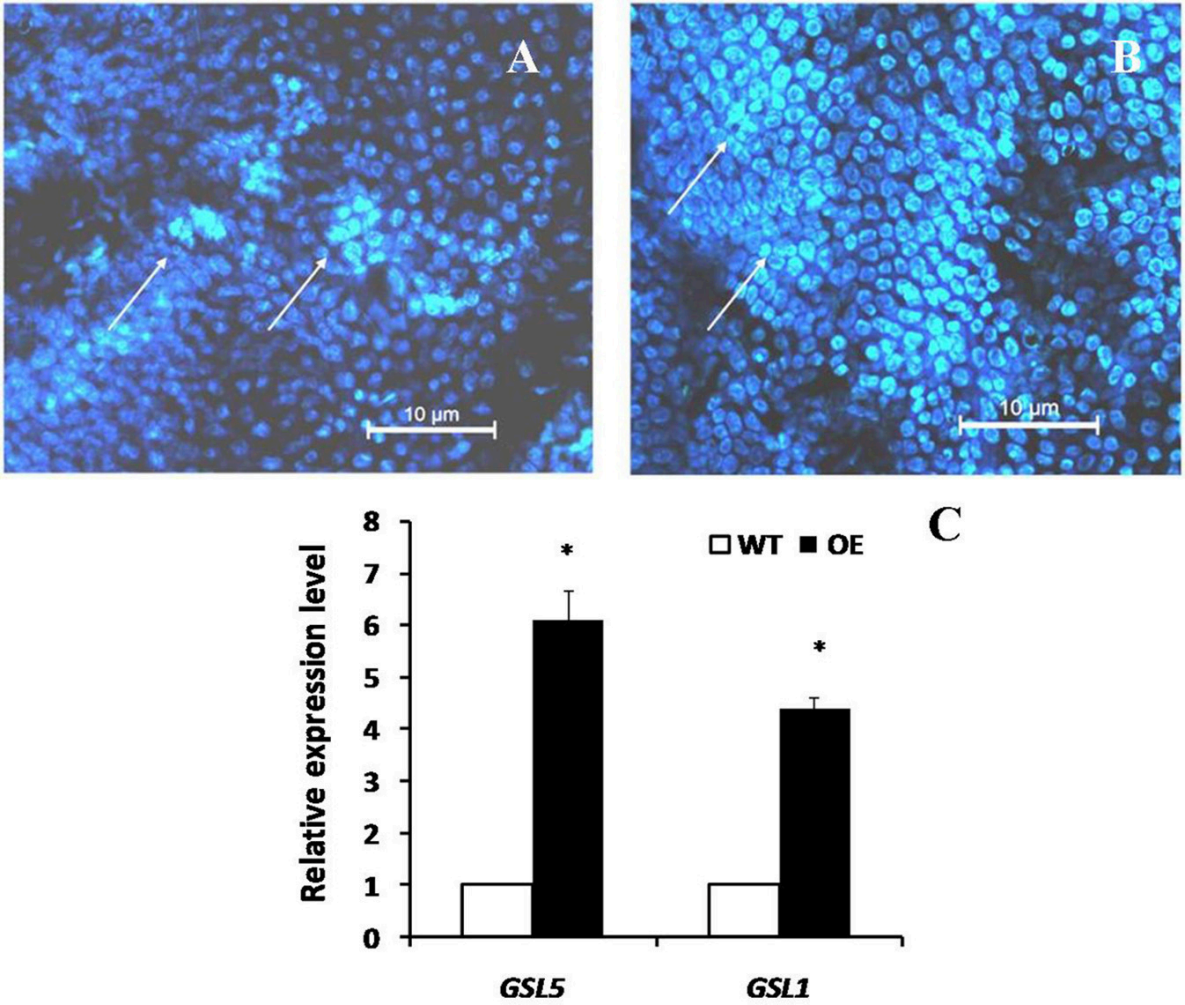
**Chromogenic assay of callose in leaves of the transgenic Arabidopsis plant and WT**. Leaves of 4 weeks old were stained for 4 h in darkness with aniline blue solution and deposition of callose in leaves was observed under fluorescence microscope of ultraviolet excitation. **(A)** Control (WT) **(B)** Transgenic Plant **(C)** Expression analysis of plant callose-related genes in transgenic (OE) and WT plants. Two callose synthase-encoding genes, *GSL1* and *GSL5* were analyzed. The *AtActin8* gene was used as reference control. The data are the means ± SE from three independent experiments. The asterisks indicate statistically significant differences between the transgenic and control plants (ANOVA followed by Tukey's tests, *P* < 0.05). Callose deposition were shown by arrows.

**Figure 5:**
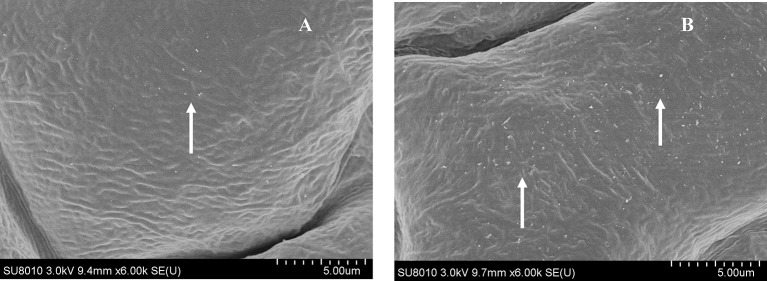
**Field Emission Scanning Electron Microscopy (FESEM) analysis of cuticle wax depositions**. Adaxial side of 4-week-old Arabidopsis rosette leaves of WT and OE were observed under 6000 × magnification. **(A)** WT showed only little wax deposition on leaf surface. **(B)** The leaf surfaces of transgenic plants with high wax deposition. Wax crystals were shown by arrows.

Plant callose is strongly related with plant resistance/tolerance to insect pests and pathogens (Jacobs et al., 2003; Hao et al., 2008; Zhang et al., 2014). Callose is recognizable in tissue sections through the formation of an intense yellow, UV light-induced fluorescence with the aniline blue fluorochrome (Stone et al., 2003). The experimental results revealed that the fluorescence in leaves of OE plant was stronger than that of WT (Figures 4A,B), suggesting that *OsLCB2a1* over-expression may increase callose deposition to improve plant tolerance to stress. We also examined the expression patterns of callose synthase-encoding genes using qRT-PCR. Two callose synthase-encoding genes (*GSL1, GSL5*) were clearly up-regulated in OE Arabidopsis plants compared to WT (Figure 4C). Additionally we examined the leaf cuticular wax deposition, which play important roles in plant resistance to various biotic and abiotic stresses (Zhang et al., 2007; Wang et al., 2011; Ni et al., 2016). The experimental results revealed that the wax deposition was higher in leaf of OE than WT plants (Figure 5).

### Overexpression of *OsLCB2a1* inhibits aphid infestation of Arabidopsis plants

**Figure 6:**
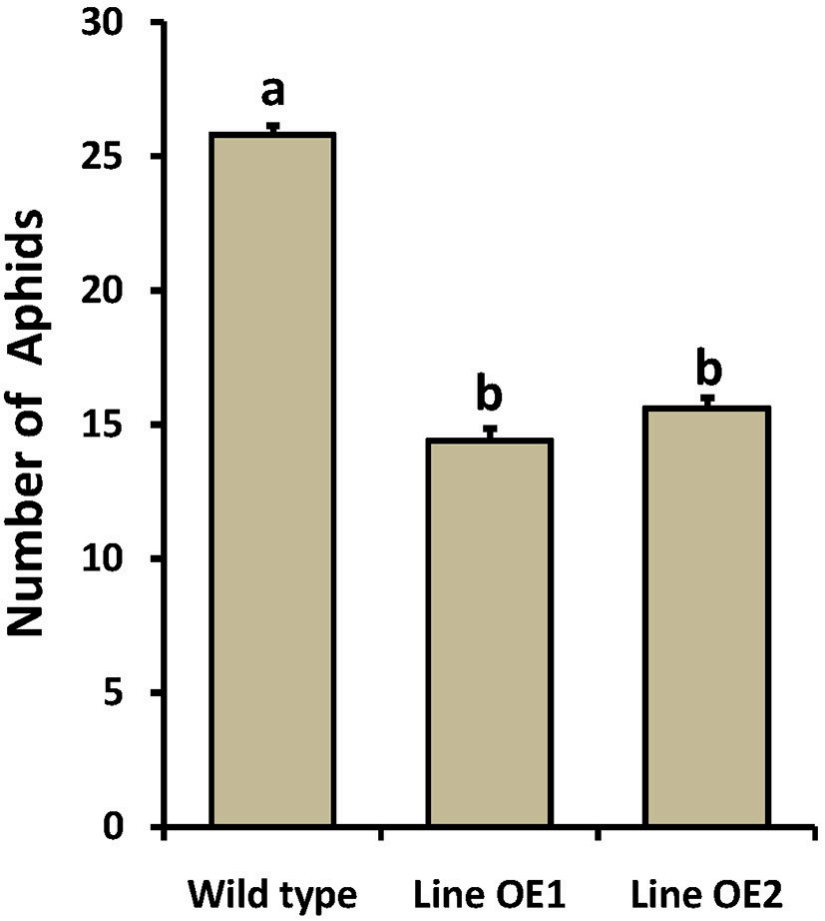
**Population density of the green peach aphid in WT and OE Arabidopsis plants**. Values represent means ± SE from 15 independent experiments. Different letters indicate significant differences (*P* < 0.05) among the treatments.

As *OsLCB2a1* expression was up-regulated in rice plant under BPH attack, we inferred that *OsLCB2a1* up regulation may improve plant resistance to aphids. To test this hypothesis, no choice assay was carried out in WT and OE Arabidopsis plants. The results showed that population size of the green peach aphids (*M. persicae*) in OE plants (Line OE1, Line OE2) was smaller than that in WT plant after 14 days (Figure 6). These results suggest that over-expression of *OsLCB2a1* might improve plant resistance to aphid.

### Overexpression of *OsLCB2a1* alters aphid probing and feeding behavior

To reveal whether aphid feeding behavior was affected by the over-expression of *OsLCB2a1* gene, we compared electrical penetration graph (EPG) recordings of *M*. *persicae* on WT or OE plants. The EPG parameters for our study are summarized in Table 1. All aphids started to penetrate the leaf around the same time on all tested plants, as indicated by the time to the first probe. Compared to the WT plants, *M. persicae* on overexpressed plant needed 1.6 times more time to the first probe to first phloem contact. Aphids on the OE plant spent significantly less time and took smaller number of time salivating into the ingesting phloem and sustain phloem sap than aphids on WT plant types (Table 1). Additionally, aphid showed a significantly longer duration of the non-probing phase on the OE than on the WT. Waveform F, associated with derailed stylet penetration, was also observed for a significantly longer time and in a larger number on the OE Arabidopsis plant (Table 1). On the other hand, the aphids spent less time taking up xylem sap from the OE plant as was indicated by a shorter time and smaller number of waveform G. Furthermore, *M. persicae* showed a significantly longer duration and larger number of potential drop phase on the OE plant than the WT plant.

**Table 1:** **Electrical penetration graph (EPG) parameters considered and their relation to ***Myzus persicae*** feeding activity on ***Arabidopsis thaliana*****.

**Related Tissue/phase**	**EPG parameter**	**WT**	**OE**
Epidermal	Time to first probe (min)	3.6±0.1a	3.7±0.0a
	Time from 1st probe to 1st phloem contact (min)	87.3±1.3b	135.6±0.7a
Phloem	Total time of phloem salivation (min)	9.9±0.2a	8.3±0.1b
	Number of phloem salivation events	7.6±0.5a	2.7±0.2b
	Average duration of phloem salivation (min)	0.9±0.0a	0.8±0.0a
	Total time of phloem ingestion (min)	104.1±1.4a	24.5±0.2b
	Number of phloem ingestion events	7.0±0.5a	1.7±0.1b
	Average duration of phloem ingestion (min)	25.3±0.6a	12.7±0.30b
	Total time of sustained (>10 min) phloem ingestion	103±9.4a	20.4±2.5b
	Number of sustained (>10 min) phloem ingestion	3.9±0.6a	1.2±0.3b
	Average duration of sustained (>10 min) phloem ingestion	25.4±1.5a	15.7±1.7b
All tissues	Total time of non-probing (NP) (min)	67.2±0.4b	98.3±1.3a
	Number of NP	38.7±0.4b	58.4±0.9a
Potential drops (PD)	Total time of PD (min)	13.9±0.6b	17.8±0.7a
	Number of PD	182.7±1.3b	442.6±1.4a
Derailed Stylet (F)	Total time of F (min)	14.0±3.5b	42.94±0.4a
	Number of F	0.5±0.1b	3.5±0.3a
Xylem (G)	Total time of G (min)	61.2±1.2a	14.8±3.3b
	Number of G	1.3±0.3b	1.0±0.2b

*Values represent means ± SEs from 15 independent experiments. Different letters indicate significant differences (P < 0.05) between the treatments*.

### Treatment with exogenous phytosphingosine increases endogenous sphingolipids and inhibits aphid infestation in Arabidopsis plants

**Figure 7:**
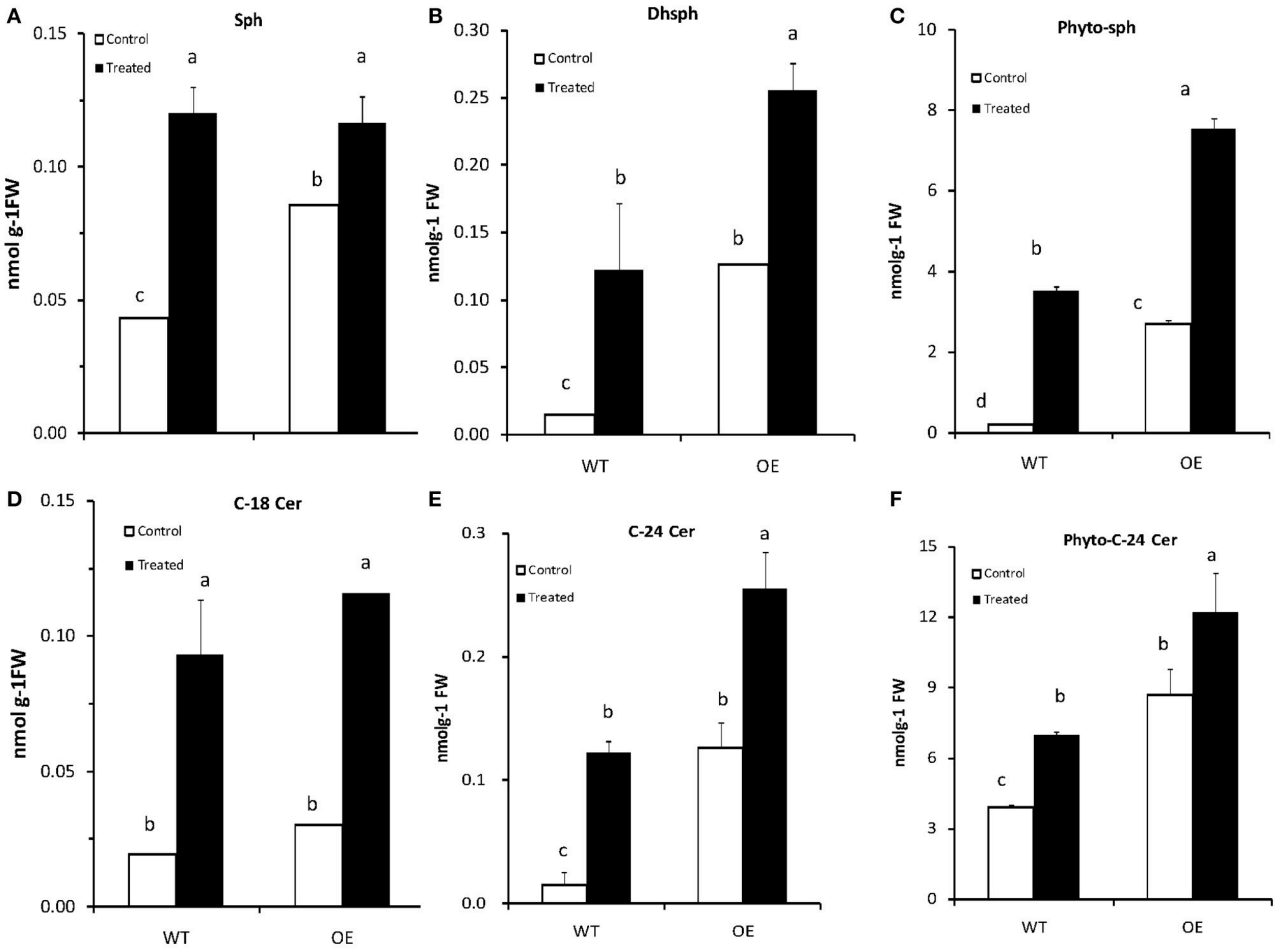
**(A–F)** Content of (nmolg^−1^FW) different LCBs and Ceramides in Arabidopsis after treated with phytosphingosine. Four-week-old plants were sprayed with phytosphingosine or methanol as control (solvent of phytosphingosine) for 8 h. Values are expressed as the mean ± SE of three independent experiments. Different letters indicate significant differences (*P* < 0.05) among the treatments (Arabidopsis and phytosph treatments). Sph, sphingosine; Dhsph, dihydrosphingosine; Cer, ceramide; FW, fresh weight.

Phytosphingosine (phyto-sph) is an intermediate in sphingolipid metabolism pathway (Figure S5) and this LCB implicated as a secondary messenger in vital signaling process in eukaryotic organism. Phyto-sph is abundant in fungi and plants, and also found in animals including humans. To find out whether exogenous insertion of sphingiod base can change the sphingolipid profile in Arabidopsis plant, we sprayed phyto-sph onto the rosette leaves of WT Arabidopsis plants and observed the sphingolipid profile. Results revealed that exogenous phyto-sph can change the sphingolipid profile in the WT plant. Figure 7 showed that the amount of three LCBs e.g., sphingosine (sph), dihydrosphingosine (Dhsph) and phyto-sph were increased significantly in the WT Arabidopsis (two-factor ANOVA, *P* < 0.05) (Figures 7A–C). The amount of C-18 Cer, C-24 Cer and phyto-24 Cer were also increased after treated with exogenous phyto-sph (Figures 7D–F).

**Figure 8:**
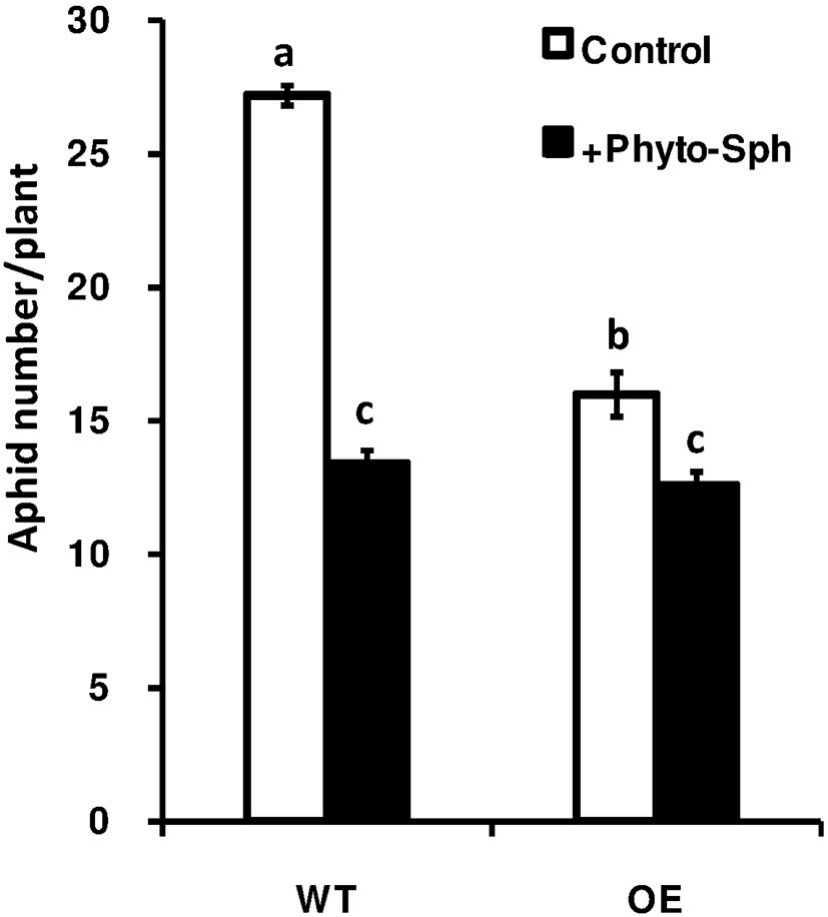
**Population density of the green peach aphid in phyto-sph treated WT and over-expressed Arabidopsis plants**. Values represent means ± SE from five independent experiments. Different letters indicate significant differences among the treatments (two-factor ANOVA, *P* < 0.05).

To find out whether the change in sphingolipid profile due to the exogenous phyto-sph in Arabidopsis plants has any impact on aphid population growth we again carried out the no choice assay in phyto-sph treated and untreated plants and results showed that population size of the green peach aphids in phyto-sph treated WT plants were reduced after 14 days compared to the control plants (Figure 8). Taken together, these results indicated that exogenous phyto-sph could inhibit aphid infestation.

### SA-dependent signaling pathway was regulated by *OsLCB2a1* in transgenic Arabidopsis under aphid attack

**Figure 9:**
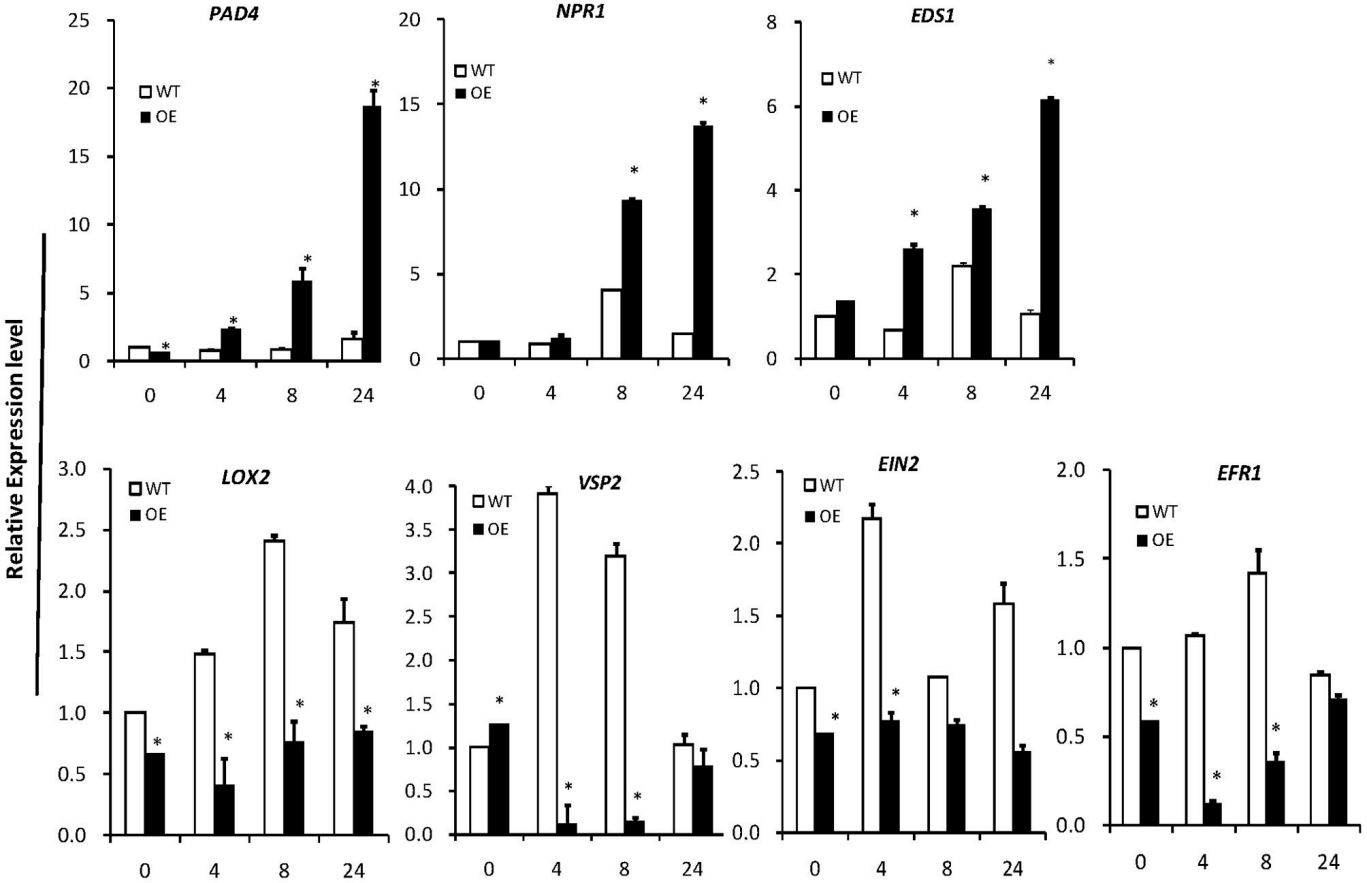
**Expression patterns of plant defense-response genes**. *PAD4, EDS1*, SA synthesis-related genes *NPR1* is a key regulator of SA-dependent systemic acquired resistance. *LOX2* and *VSP2* are the JA synthesis-related genes. *EIN2* and *EFR1* is the ethylene signaling pathway receptor gene. *AtActin8* was used as reference control. In all panels, the mean is based on the average of three independent experiments. One-way ANOVA was used to generate the *P*-values (^*^P < 0.05).

To investigate the signal transduction pathway involved in *OsLCB2a1*-mediated aphid resistance, we examined the expression levels of some defense-responsive genes, which are known to function in SA- and JA/ethylene-dependent pathways (Li et al., 2006; Zarate et al., 2007). Expression levels of the genes *EDS1* (*ENHANCED DISEASE SUSCEPTIBILITY1*), *PAD4* (*PHYTOALEXIN DEFICIENT 4*), *NPR1* (*NONEXPRESSOR* OF *PATHOGENESIS-RELATED GENES 1*) related to SA-dependent pathway were remarkably higher in the OE plants than that in WT at 0, 4, 8, and 24 h under aphid attack (Figure 9). However, the transcripts of *LOX2* (*LIPOXYGENASE 2*), *VSP2* (*VEGETATIVE STORAGE PROTEIN 2*), *EIN2* (*ETHYLENE INSENSITIVE 2*) and *ERF1* (*ETHYLENE RESPONSE FACTOR 1*) on JA/ethylene-dependent pathway were significantly lower in OE lines than those in WT (Figure 9). The results suggest that *OsLCB2a1* might mediate the transgenic resistance to aphids through SA-dependent signaling.

## Discussion

Sphingolipids are indispensable components of eukaryotic cells, which play an important role in signal transduction pathways by regulating physiological functions and influencing cell behavior. Recent studies have noted the diversity of sphingolipid functions during plant development and stress responses. In plants, LCBs act as bioactive molecules in the immune response (Rivas-San Vicente et al., 2013). In this study, we have characterized the rice LCB2a gene encoding a subunit of SPT. Multiple alignment showed that *OsLCB2a* has a conserved GTFTKSFG motif corresponding to the known PLP-binding site like *N. banthamiana, A. thaliana, S. cerevisiae* and *Homo sapiens* (Figure 1A). Takahashi et al. (2009) hypothesized that over-expression of *NbLCB2* enhanced the SPT activity which in turn triggered plant cell death. Such speculations offer valuable clues to uncover the function of SPT in herbivore resistance.

In this study, we found that the *OsLCB2a1*-overexpressing plants accumulated more LCBs and ceramides. As we know that SPT is the enzyme that catalyzes the first reaction in sphingolipid metabolism we hypothesized that over-expression of the LCB2 subunit caused a general increase in LCBs levels, especially of sphinganine. Sphinganine is the first LCB synthesized in this pathway and is a substrate for desaturases and hydroxylases to generate the other LCBs (Chen et al., 2009). However, we found that the trihydroxylated LCB phytosphingosine (t18:0), which is more abundant, increased 1.7 fold and phytoceramide increased 1.3 fold in over-expressed plant compared to WT. Whereas, levels of the dihydroxylated LCBs sphinganine (d18:0) decreased and sphingosine (d18:1) remained unchanged. This differential effect of SPT over-expression on LCBs profile revealed the complexity of the LCBs metabolism and regulation which is in agreement with the results from Chen et al. (2006) and Rivas-San Vicente et al. (2013). We also found that the amount of ceramide specially phytoceramide and ceramide also increased in *OsLCB2a1*-overexpressing plants. These data suggest that *OsLCB2a1* regulates sphingolipid homeostasis, and that a complex regulatory network of sphingolipid metabolism exists in plants.

Plants have evolved a variety of defense and tolerance mechanisms against insect herbivore during the interactions of plants with insect pests. Plant callose is not only important for the normal growth and metabolism of plant, but also is closely related to plant resistance to insect pests and pathogens. Similarly, wax deposition also plays an important role in plant resistance to various biotic and abiotic stresses. Our study revealed that *OsLCB2a1*-overexpressing Arabidopsis plants contained more callose and wax compared to WT (Figures 4, 5), suggesting that the increase in callose and wax content might improve the transgenic plants tolerance to aphid attack. No-choice aphid assay showed that population size of the green peach aphid (GPA, *M. persicae*) in *OsLCB2a1*-overexpressing plants was smaller than that in WT plant after 14 days, indicating that, *OsLCB2a1* might improve the plant attribute to cope with the herbivore attack. This observation is in agreement with many reports that show that over-expression of certain gene enhances resistance to herbivore. For instance over-expression of *Increased Resistance to Myzus persicae 1* (*IRM1*) gene might result in mechanical barriers that reduces the population size of *M*. *persicae* in *A. thaliana* (Chen et al., 2013). Likewise, the aphid population size was smaller in transgenic plants, overexpression of cotton photosynthesis-related gene *GhPSAK1* suggesting that *GhPSAK1* might improve aphid-tolerance of the transgenic plants (Zhang et al., 2014). Over-expression of the PAP1 transcription factor in tobacco, responsible for accumulation of anthocyanin pigments and other flavonoids/ phenylpropanoids exhibited greater resistance to the herbivore insect, *Spodoptera litura* (Mitsunami et al., 2014). Xin et al. (2014) showed that over-expression of a xylanase inhibitor gene, *OsHI-XIP* decreased the feeding and oviposion preferences of the rice BPH, *N*. *lugens*.

Plants have evolved several defense strategies against damage caused by herbivores including antibiotic factors restricting insect fecundity and anti-xenotic factors deterring insects from settling on the host and feeding. Information on the aphid activities can be extracted from the recorded signal waveforms through EPG that provides insight into the location of plant resistance factors. Our EPG results revealed that over-expression of *OsLCB2a1* gene affected the aphids in its ability to penetrate the phloem. The results of other parameters also support this statement. For instance the phloem phase of aphid on WT was faster and sustained long time compared to *OsLCB2a1*-overexpressing plants. Moreover, the aphid spent significantly shorter time in the xylem of *OsLCB2a1*-overexpressing plants than on that of WT plants. The over-expression of *OsLCB2a1* reduced the number of sustained phloem sap ingestion indicating an enhanced phloem-based resistance. These findings can be supported by the relevant previous studies. For example Chen et al. (2013) hypothesized that over-expression of *IRM1* interrupted the capability of *M. persicae* to reach sustained phloem sap ingestion and the tested aphids needed double the time compared to the WT. Over-expression of *SKU5 SIMILAR 13* (*SKS13*) in *A. thaliana* led to a reduced phloem feeding of *M. persicae*, which was probably due to accumulation of ROS in leaves. Eventually the reduced phloem feeding resulted in the suppression of the population development of *M. persicae* (Chen et al., 2014). Zhu et al. (2011) reported that the soybean aphid Aphis glycines spent significantly shorter periods of time in the sieve element phase but slightly more times in non-probing phases in antibiotic resistant lines of soybean than in the susceptible control suggesting an existence of resistance factors in the phloem of the resistant soybean lines. Green peach aphid spent more time in active feeding from the sieve elements of *phytoalexin deficient4* (*PAD4*) mutants than from WT plants, and less time feeding on transgenic plants in which PAD4 was ectopically expressed (Pegadaraju et al., 2007).

As we found that the *OsLCB2a1*-overexpressing plants accumulated more LCBs and ceramides, we hypothesized that exogenous insertion of sphingiod base can also change the sphingolipid profile in plants and as well as have impact on insect. Results revealed that exogenous phyto-sph can change the sphingolipid profile in WT plants (Figure 7) and the treated plants have negative impact on aphid population build up suggesting that sphingoipid is involved in plant response to insect attack (Figure 8).

Plant defense response to insect is mediated by various signaling molecules including phytohormones. As we noticed significant upregulation in *OsLCB2a* expression upon BPH attack (Figure 2), we speculated a potential involvement of the SA- and/or JA-dependent signaling pathway in *OsLCB2a* overexpression-promoted insect resistance in plants. Our results suggest that *OsLCB2a1* may activate some defense response genes on the SA-dependent signaling pathway and inhibit some genes on JA/ethylene-dependent signaling pathways (Figure 9). Likewise, a previous study revealed that BPH14 gene confers rice resistance to BPH (Du et al., 2009) and cotton photosynthesis-related gene (*GhPSAK1*) enhanced SA signaling pathway in transgenic plants (Zhang et al., 2014). Sánchez-Rangel et al. (2015) reviewed that sphingolipid played a key role in defense against pathogen in SA-dependent pathway.

Taken together, our results showed that over-expression of *OsLCB2a1* increased LCBs and ceramides levels in transgenic plants. In addition, exogenous application of sphingolipid metabolite could also increase the amount of LCBs and ceramides in Arabidopsis plants. *OsLCB2a1* overexpressing transgenic plants accumulated higher levels of leaf callose and wax and had a small population size of *M. persicae*. Electrical monitoring of aphid feeding behavior revealed that *OsLCB2a1* modulated a phloem-based defense mechanism against the aphid. It is plausible that *OsLCB2a1* overexpression resulted in increased ceramides and LCBs by affecting sphingolipids biosynthesis and thus influencing the protein that are encoded by *OsLCB2a1* to regulates plant responses to biotic and abiotic stresses. Further studies on proteomics may unveil the molecular mechanism of *OsLCB2a1*–mediated plant defense involving sphingolipid biosynthesis.

## Author contributions

MB contributed to the experimental design, performed the experiments and wrote the manuscript. XS and YT performed part of the experiments. CM contributed to the experimental design and critical revision of the manuscript. ZZ planned the study. All the authors revised manuscript.

### Conflict of interest statement

The authors declare that the research was conducted in the absence of any commercial or financial relationships that could be construed as a potential conflict of interest.
