# Supplementary material for: Molecular Characterization of Rice OsLCB2a1 Gene and Functional Analysis of its Role in Insect Resistance
Source: Front Plant Sci. 2016 Dec 1;7:1789. doi: 10.3389/fpls.2016.01789 (PMC5130998; doi:10.3389/fpls.2016.01789)
Supplement: Supplementary file 1 [file Table1.docx]

Table S1: Accession numbers of different organism used in the tree contraction in the NCBI and Phytozome Genbank

| **Organism name and protein ID** | **Accession number** |
| --- | --- |
| *Arabidopsis thaliana* (AtLCB2a) | AT5G23670.1 |
| *Aspergillus oryzae* (AoLCB2a.1) | XP_001818901.1 |
| *Aspergillus oryzae* (AoLCB2a.2) | XP_001816940.1 |
| *Brachypodium distachyon* (BdLCB2a.1) | Bradi2g59530.1 |
| *Brachypodium distachyon* (BdLCB2a.2) | Bradi4g18210.1 |
| *Clamydomonas reinhardtii* (CrLCB2a) | g5038.t1 |
| *Citrus sinensis* (CsLCB2a.1) | orange1.1g046230m |
| *Citrus sinensis* (CsLCB2a.2) | orange1.1g040324m |
| *Drosophila melanogaster* (DmLCB2a.1) | NP_476614.1 |
| *Drosophila melanogaster* (DmLCB2a.2) | BAA83721.1 |
| *Eucalyptus grandis* (EgLCB2a) | Eucgr.K02201.1 |
| *Glycine max* (GmLCB2a.1) | Glyma16g26210.1 |
| *Glycine max* (GmLCB2a.2) | Glyma02g07250.1 |
| *Homo sapiens* (HsLCB2a.1) | NP_004854.1 |
| *Homo sapiens* (HsLCB2a.2) | BAA25452.2 |
| *Homo sapiens* (HsLCB2a.3*)* | EAX10321.1 |
| *Homo sapiens* (HsLCB2a.4) | NP_060797.2 |
| *Homo sapiens* (HsLCB2a.5) | AAI50645.1 |
| *Mus musculus* (MsLCB2a.1) | NP_035609.1 |
| *Mus musculus* (MsLCB2a.2) | NP_780676.1 |
| *Mus musculus* (MsLCB2a.3) | EDL28402.1 |
| *Oryza sativa* (OsLCB2a.1) | LOC_Os01g70380.1 |
| *Oryza sativa* (OsLCB2a.2) | LOC_Os01g70370.1 |
| *Oryza sativa* (OsLCB2a.3) | LOC_Os11g31640.1 |
| *Physcomitrella patens* (PpLCB2a) | Pp1s377_35V6.1 |
| *Pseudomonas sp.*(PLCB2a) | WP_008095099.1 |
| *Sorghum bicolour* (SbLCB2a.1) | Sb03g044700.1 |
| *Sorghum bicolour* (SbLCB2a.2) | Sb05g018870.1 |
| *Sorghum bicolour* (SbLCB2a.3) | Sb05g018880.1 |
| *Selaginella moellendorffii* (SmLCB2a) | 86377 |
| *Solanum lycopersicum* (SlLCB2a.1) | Solyc06g030590.1.1 |
| *Solanum lycopersicum*(SlLCB2a.2) | Solyc03g098220.2.1 |
| *Solanum lycopersicum* (SlLCB2a.3) | Solyc06g072320.2.1 |
| *Vitis Vinifera* (VvLCB2a.1) | GSVIVT01033008001 |
| *Vitis Vinifera* (VvLCB2a.2) | GSVIVT01008621001 |
| *Zea mays* (ZmLCB2a.1) | GRMZM2G010202_T01 |
| *Zea mays* (ZmLCB2a.) | GRMZM2G142030_T01 |
| *Zea mays* (ZmLCB2a.3) | GRMZM2G152888_T01 |
| *Zea mays* (ZmLCB2a.4) | GRMZM2G357734_T03 |
| *Zea mays* (ZmLCB2a.5) | GRMZM2G067257_T01 |
| *Zea mays* (ZmLCB2a.6) | GRMZM2G055854_T01 |
| *Populus trycocarpa* (PtLCB2a.1) | Potri.015G103800.1 |
| *Populus trycocarpa* (PtLCB2a.2) | Potri.012G104500.1 |
| *Nicotiana banthamiana* | (NbLCB2.1) AB378334.1 |
| *Nicotiana banthamiana* | (NbLCB2.2) ABW97709.1. |

**Table S2 Primers used in the study**

| Primers name | Primers Sequence (5^ʹ^……………………..3^ʹ^ ) |
| --- | --- |
| 70-F | TCTATCTCCGCATCCAGGAC |
| 70-R | ACACGAGGGGTGCAGTATTC |
| \| OsActinF \|  \| \| --- \| --- \| | CAGCACATTCCAGCAGAT |
| OsActinR | GGCTTAGCATTCTTGGGT |
| T-DNA RP | TGTGAATGCAATTCCAACATG |
| T-DNA LP | ACGCCACATCAATTTCAACTC |
| LBb1.3 | ATTTTGCCGATTTCGGAAC |
| At5g23670_F | TCTACTGCTGGCTAGGGCA |
| At5g23670_R | GACTGTTCTATTTTCTTCGGCT |
| Actin_7 F | CCATTCAGGCCGTTCTTTC |
| Actin_7 R | CGTTCTGCGGTAGTGGTGA |
| Oslcb2_F | ACTGCATCTGTTGAGAAACTG |
| Oslcb2_R | CAGTGTCTATCATTGTGTTGT |
| LBSP1 | TTTCTCCATAATAATGTGTGAGTAGTTCCC |
| LBSP2 | CTCATGTGTTGAGCATATAAGAAACCCTTAG |
| LBSP3 | CTAAAACCAAAATCCAGTACTAAAATCC |
| AD1 | NTCGASTWTSGWGTT |
| AD2 | NGTCGASWGANAWGAA |
| AD3 | WGTGNAGWANCANAGA |
| OE LB500 | CATACGCTAGTTCCAACCAGAATC |
| OE RB1000 | GCTAACAGTCTTCCATCAGTTTCC |
| RB3 | AGCTTGAGCTTGGATCAGATTGTCGT |
| Actin8_F | GATGGAGACCTCGAAAACCA |
| Actin8_R | AAAAGGACTTCTGGGCACCT |
| GSL1R | AGTCCGGCCAAAGTAATGGG |
| GSL1F | CTCTCGAGCGTGGGTTTCTT |
| GSL5F | TGCATTCATCCCAACTGGCT |
| GSL5R | ACATGCGAGCAACAGAGACA |
| LOX2 F | AGATTCAAAGGCAAGCTCCA |
| LOX2 R | ACAACACCAGCTCCAGCTCT |
| VSP2 F | TACGAACGAAGCCGAACTCT |
| VSP2R | GGCACCGTGTCGAAGTCTAT |
| PAD4 F | GTTCTTTTCCCCGGCTTATC |
| PAD4 R | CGGTTATCACCACCAGCTTT |
| EDS1F | TCGAAG GGGACATAG ATTGG |
| EDS1R | CTTTTCATGTACGGCCCTGT |
| NPR1F | TCACTG GTACGAAGAGAACA |
| NPR1R | TGAGAGAGTTTACGGTTA |
| ERF1F | CTTCCGACGAAGATCGTAGC |
| ERF1R | TCTTGACCGGAACAGAATCC |
| EIN2F | GGTTTGAGATGGAATACCGTGATGG |
| EIN2R | TCAAGGATGGCAGATAAGTGTCTCC |
