# Supplementary material for: Molecular Characterization of Rice OsLCB2a1 Gene and Functional Analysis of its Role in Insect Resistance
Source: Front Plant Sci. 2016 Dec 1;7:1789. doi: 10.3389/fpls.2016.01789 (PMC5130998; doi:10.3389/fpls.2016.01789)
Supplement: Supplementary file 2 [file Table2.docx]

**Table S2 Primers used in the study**

| Primers name | Primers Sequence (5^ʹ^……………………..3^ʹ^ ) |
| --- | --- |
| 70-F | TCTATCTCCGCATCCAGGAC |
| 70-R | ACACGAGGGGTGCAGTATTC |
| \| OsActinF \|  \| \| --- \| --- \| | CAGCACATTCCAGCAGAT |
| OsActinR | GGCTTAGCATTCTTGGGT |
| T-DNA RP | TGTGAATGCAATTCCAACATG |
| T-DNA LP | ACGCCACATCAATTTCAACTC |
| LBb1.3 | ATTTTGCCGATTTCGGAAC |
| At5g23670_F | TCTACTGCTGGCTAGGGCA |
| At5g23670_R | GACTGTTCTATTTTCTTCGGCT |
| Actin_7 F | CCATTCAGGCCGTTCTTTC |
| Actin_7 R | CGTTCTGCGGTAGTGGTGA |
| Oslcb2_F | ACTGCATCTGTTGAGAAACTG |
| Oslcb2_R | CAGTGTCTATCATTGTGTTGT |
| LBSP1 | TTTCTCCATAATAATGTGTGAGTAGTTCCC |
| LBSP2 | CTCATGTGTTGAGCATATAAGAAACCCTTAG |
| LBSP3 | CTAAAACCAAAATCCAGTACTAAAATCC |
| AD1 | NTCGASTWTSGWGTT |
| AD2 | NGTCGASWGANAWGAA |
| AD3 | WGTGNAGWANCANAGA |
| OE LB500 | CATACGCTAGTTCCAACCAGAATC |
| OE RB1000 | GCTAACAGTCTTCCATCAGTTTCC |
| RB3 | AGCTTGAGCTTGGATCAGATTGTCGT |
| Actin8_F | GATGGAGACCTCGAAAACCA |
| Actin8_R | AAAAGGACTTCTGGGCACCT |
| GSL1R | AGTCCGGCCAAAGTAATGGG |
| GSL1F | CTCTCGAGCGTGGGTTTCTT |
| GSL5F | TGCATTCATCCCAACTGGCT |
| GSL5R | ACATGCGAGCAACAGAGACA |
| LOX2 F | AGATTCAAAGGCAAGCTCCA |
| LOX2 R | ACAACACCAGCTCCAGCTCT |
| VSP2 F | TACGAACGAAGCCGAACTCT |
| VSP2R | GGCACCGTGTCGAAGTCTAT |
| PAD4 F | GTTCTTTTCCCCGGCTTATC |
| PAD4 R | CGGTTATCACCACCAGCTTT |
| EDS1F | TCGAAG GGGACATAG ATTGG |
| EDS1R | CTTTTCATGTACGGCCCTGT |
| NPR1F | TCACTG GTACGAAGAGAACA |
| NPR1R | TGAGAGAGTTTACGGTTA |
| ERF1F | CTTCCGACGAAGATCGTAGC |
| ERF1R | TCTTGACCGGAACAGAATCC |
| EIN2F | GGTTTGAGATGGAATACCGTGATGG |
| EIN2R | TCAAGGATGGCAGATAAGTGTCTCC |
