# Supplementary material for: Molecular Characterization of Rice OsLCB2a1 Gene and Functional Analysis of its Role in Insect Resistance
Source: Front Plant Sci. 2016 Dec 1;7:1789. doi: 10.3389/fpls.2016.01789 (PMC5130998; doi:10.3389/fpls.2016.01789)
Supplement: Supplementary file 3 [file DataSheet1.pdf]

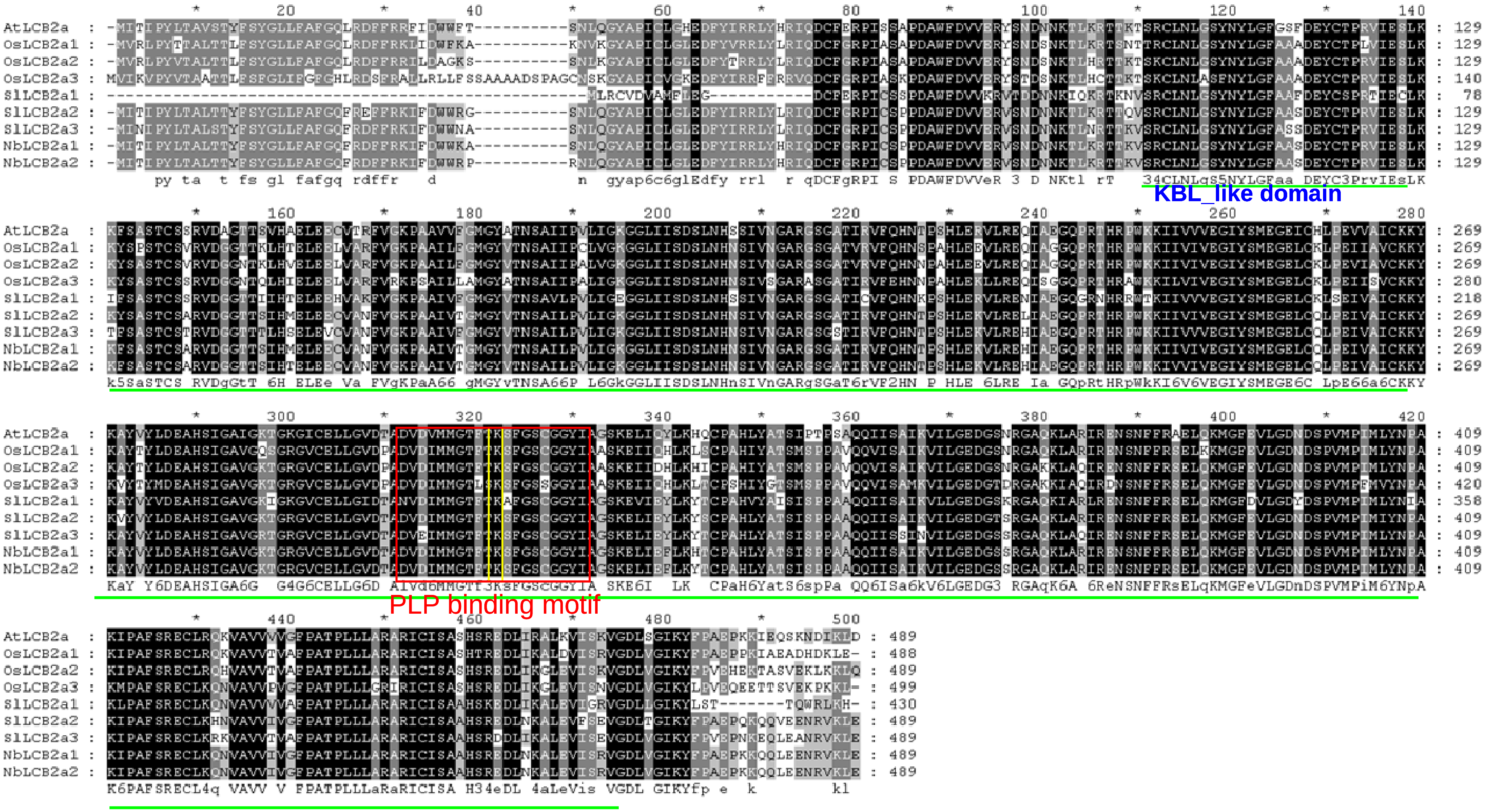

Figure S1. Alignment of deduced amino acid sequence of LCB2 from *Arabidopsis thaliana*, *Oryza sativa Solanum lycopersicum* And *Nicotiana banthamiana*

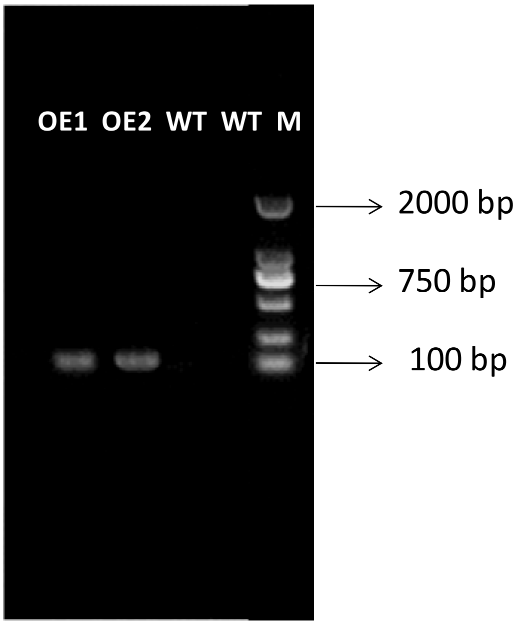

Figure S2. RT-PCR identification of Transgenic *Arabidopsis thaliana* M: DNA marker DL2000; OE=Over-expression, WT= Wild type

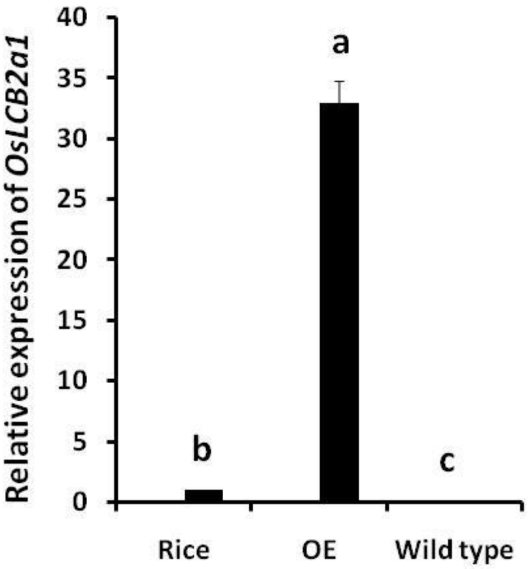

Figure S3. Quantification of *OsLCB2a1* expression in 4-week-old Arabidopsis leaves. Total RNA was extracted for qRTPCR analysis. *OsActin* was use as an internal control. Gene expression values are presented relative to average rice levels (Set as 1). Data represent the mean ± SE from three replicates. Data set marked with different letters indicate significant difference assessed by Tukey's test ($p < 0.05$).

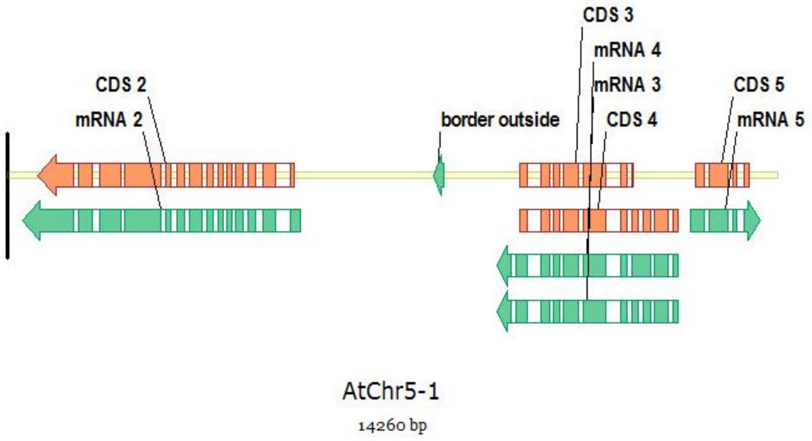

Figure S4. Blast result of T-DNA insertion of OE plant. The insertion site located upstream 1kb of AT5G56900 (CDS3/4/5) and downstream 2.7kb of AT5G56890 (CDS2) without any annotated genes broken in the OE plant

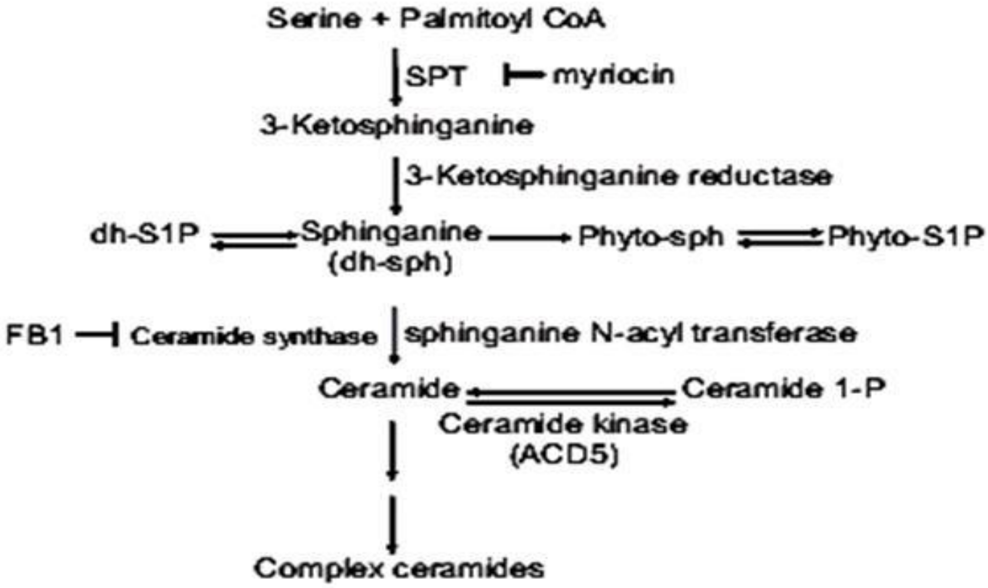

Figure S5. A proposed pathway of plant sphingolipid *de novo* synthesis. Myriocin and FB1 are competitive inhibitors of SPT and ceramide synthase, respectively (Shi et al., 2007)
